# Kaempferol Restores Cilia Length Through Axonemal Microtubule Stabilization

**DOI:** 10.3390/cells15191806

**Published:** 2026-10-03

**Authors:** Xuyao Dai, Yi Zhao, Ruodan Xu, Ning Li

**Affiliations:** Department of Biomedical Engineering and Technology, Institute of Basic Theory for Chinese Medicine, China Academy of Chinese Medical Sciences, Beijing 100700, China; daixuyao000@163.com (X.D.); zy93607@163.com (Y.Z.)

**Keywords:** cilia, ciliary length, ciliopathy, kaempferol, microtubule stability, acetylated α-tubulin

## Abstract

**Highlights:**

**What are the main findings?**

- Kaempferol promotes cilia elongation under basal and physiological conditions.
- Kaempferol restores cilia length and enhances axonemal microtubule stability across distinct defect and stress models.

**What are the implications of the main findings?**

- Axonemal microtubule stabilization serves as a key mechanism for ciliary length maintenance and restoration.
- Kaempferol and microtubule-stabilizing strategies offer potential for ciliary-associated disorders.

**Abstract:**

Ciliary shortening disrupts cilium-dependent signaling and tissue homeostasis and contributes to diverse ciliopathies and acquired disorders. However, the underlying mechanisms and pharmacological strategies for restoring ciliary length remain poorly understood. Here, we identify Kaempferol (Kae) as a previously unrecognized small-molecule regulator of ciliary length. Transcriptomic profiling showed that Kae-responsive genes were enriched in cytoskeleton- and microtubule-related processes, including microtubule organization and motor protein activity, suggesting that cytoskeletal regulation may contribute to its effects on ciliary length. Further investigation demonstrated that Kae promoted ciliary elongation under basal conditions and restored ciliary length across mechanistically distinct defect models, including SARS-CoV-2 non-structural protein 13 (NSP13) expression, Ciliobrevin D-mediated dynein inhibition, and centrosomal protein 290 (CEP290) deficiency. Mechanistically, Kae enhanced microtubule stability, as indicated by increased acetylated α-tubulin (ACTUB) expression, greater resistance to nocodazole-induced depolymerization, and enhanced microtubule recovery. Consistent with these cellular effects, Kae promoted microtubule recovery and preserved ciliary ultrastructure in the NSP13-induced airway injury model in vivo. These findings establish Kae as a pharmacological modulator of ciliary length and highlight axonemal microtubule stabilization as a potential strategy for restoring dysfunctional cilia.

## 1. Introduction

Cilia are evolutionarily conserved, microtubule-based, antenna-like sensory organelles that protrude from the surface of most mammalian cells, functioning as critical signaling platforms for sensing and integrating extracellular signals [1]. Characterized by microtubule-based axonemes with canonical “9 + 0” or “9 + 2” configurations, cilia regulate a wide range of cellular processes, including development, differentiation, proliferation, and tissue homeostasis [2]. Structurally, cilia are composed of a mother-centriole-derived basal body, a transition zone, a ciliary membrane, and a central microtubule backbone known as the axoneme [3]. Ciliogenesis and ciliary maintenance require a tightly coordinated, two-step process: ciliary initiation through basal body docking and ciliary vesicle recruitment, and ciliary elongation by intraflagellar transport (IFT)-mediated trafficking and axonemal microtubule assembly [4]. Axonemal stability relies heavily on post-translational modifications, particularly the acetylated α-tubulin (ACTUB), which confers mechanical resilience and maintains ciliary architecture [5]. Disruption of either ciliary initiation or axonemal elongation results in defective or shortened cilia, giving rise to a group of genetic disorders termed ciliopathies as well as acquired ciliary injuries induced by environmental stress or viral infections [6,7,8]. Thus, identifying small molecules capable of regulating distinct stages of ciliary homeostasis represents a potential therapeutic strategy for treating ciliary dysfunction and associated disorders.

Despite growing interest in ciliary biology, pharmacological agents that directly target ciliogenesis or structural restoration remain scarce. Traditional studies on natural compounds, such as quercetin, platycodin D, and hesperidin, have largely focused on their secondary benefits, including antioxidant activity, suppression of nuclear factor kappa B (NF-κB)/nuclear factor erythroid 2-related factor 2 (NRF2)-mediated inflammatory cascades, or enhancement of mucociliary clearance in motile cilia [9,10,11]. However, whether natural small molecules can directly act as structural regulators of cilia by stabilizing the axoneme or reversing pathologically induced ciliary regression remains underexplored. In particular, it remains unclear whether natural therapeutics exhibit differential regulatory capacities toward ciliary initiation versus ciliary elongation under distinct physiological and stress conditions.

Kaempferol (Kae), a natural flavonol abundant in various medicinal sources, possesses versatile pharmacological activities [12]. Beyond its well-documented cytoprotective properties, emerging evidence suggests that Kae can modulate cytoskeletal dynamics and tubulin organization, processes intrinsically linked to ciliary axoneme assembly and elongation [13,14]. These traits raise the compelling hypothesis that Kae may serve as a targeted small-molecule modulator of cilia structure and stability.

In this study, to overcome limitations associated with conventional serum-starvation stress models and to evaluate broader physiological and stress relevance, we investigated the ciliary-lengthening efficacy of Kae across diverse serum conditions as well as distinct genetic and pharmacological perturbation models. Under physiological and stress conditions alike, Kae significantly promoted ciliary elongation and enhanced axonemal structural protein recovery. Notably, when tested across different injury scenarios, Kae exhibited a distinct, mechanism-dependent rescue pattern. Firstly, in a viral injury model, Kae comprehensively restored both ciliation frequency and ciliary length. In contrast, in models with structural or machinery deficits, specifically, IFT motor blockade using Ciliobrevin D and transition zone impairment by CEP290 deficiency, Kae selectively rescued ciliary length without altering initial ciliation frequency, accompanied by increased ADP-ribosylation factor-like protein 13B (ARL13B) and ACTUB levels, which is associated with promoted axonemal microtubule stability. Our findings demonstrated that Kae acts as a selective pro-elongation natural small molecule that protects cilia against stress collapse primarily by stabilizing axonemal microtubules. This work provides potential pharmacological insights into applying natural compounds for targeted ciliary structural repair.

## 2. Materials and Methods

### 2.1. Cell Culture

ARPE-19 cells (Procell, Wuhan, China) and RPE-1 cells (ATCC, Manassas, VA, USA) were maintained in DMEM/F-12 (1:1) supplemented with 10% FBS and 1% P/S at 37 °C under 5% CO_2_. MRC-5 cells (Procell) were maintained in MEM supplemented with NEAA, 10% FBS and 1% P/S at 37 °C under 5% CO_2_. Cell identity was confirmed by STR profiling.

To induce cilia formation, cells were cultured under reduced-serum conditions with 10%, 5%, 2%, or 0.5% FBS for 24 h. Unless otherwise indicated, cells were cultured in 0.5% FBS for 24 h for cilium induction.

### 2.2. Cell Transfection

Human codon optimized sequences for genes encoding SARS-CoV-2 NSP13 (Gene ID: 43740578), fused to a C-terminal FLAG-tag (peptide sequence: DYKDDDDK) was synthesized by GenScript (Nanjing, China). The small interfering RNA targeting CEP290 (si-*CEP290*) was obtained from RiboBio (Guangzhou, China). The siRNA target sequence was 5′-GGATTCGGATGAAATGAAA-3′.

ARPE-19 cells were transfected with the NSP13 plasmid using Starvio Transfection Reagent (Starvio Biotech, Shanghai, China), whereas siRNA transfection was performed with the RiboFECT™ CP Transfection Kit (RiboBio), according to the manufacturers’ protocols. At 24 h after transfection, the culture medium was replaced with serum-reduced medium (0.5% FBS) or physiological serum-containing medium (2% FBS), and cells were treated with Kaempferol (Kae, Shanghai Yuanye Bio-Technology Co., Ltd., Shanghai, China) at final concentrations of 1 μM, 3 μM, and 5 μM for an additional 3 h or 24 h.

### 2.3. Microtubule Stability and Regrowth Assay

ARPE-19 cells were pretreated with either DMSO or Kae (5 μM) in 0.5% serum-containing medium for 24 h. Following pretreatment, cells in both groups were exposed to 10 μM nocodazole (MedChemExpress, Monmouth Junction, NJ, USA) for 30 min to induce complete microtubule depolymerization. After nocodazole treatment, cells were thoroughly washed with PBS to remove residual nocodazole and subsequently incubated in 0.5% serum-containing medium to allow microtubule regrowth. Cells were fixed immediately after nocodazole removal (0 min) or after 5, 15, 30, and 60 min of recovery and subjected to immunofluorescence staining.

### 2.4. Cell Immunofluorescence Staining

Cells were seeded onto coverslips placed in 24-well plates at 1 × 10^5^ cells/mL. Following fixation with 4% paraformaldehyde (PFA, Aoqing Biotechnology, Beijing, China) for 15 min at room temperature (RT), the cells were rinsed three times with PBS, with each wash lasting 5 min. Cell membranes were permeabilized with 0.2% Triton X-100 in PBS for 20 min and subsequently blocked with 10% goat serum in PBS for 1 h at RT. The cells were then incubated with the indicated primary antibodies overnight at 4 °C. After three washes with PBS on the following day, Alexa Fluor-labeled secondary antibodies were applied for 2 h at RT. Nuclear staining was performed with 4′,6-diamidino-2-phenylindole (DAPI) for 5 min at RT. The coverslips were finally mounted onto glass slides using fluorescence mounting medium (Abcam, Cambridge, UK). Images were acquired on an Olympus FV3000 confocal microscope (Olympus Corporation, Tokyo, Japan) and processed for quantitative analysis using ImageJ (v 1.49) (National Institutes of Health, Bethesda, MD, USA). Primary antibodies included rabbit anti-ADP-ribosylation factor-like protein 13B (ARL13B, 1:1000, Proteintech, Chicago, IL, USA), mouse anti-acetylated α-tubulin (1:2000, Sigma, St. Louis, MO, USA), rabbit anti-NSP13 (1:1000, Abcam), rabbit anti-CEP290 (1:1000, Proteintech), rabbit anti-CEP164 (1:500, Proteintech), mouse anti-IFT81 (1:200, Proteintech) and mouse anti-α-tubulin (1:1000, Proteintech). Alexa Fluor 488- and Alexa Fluor 594-conjugated secondary antibodies (1:1000, Thermo Fisher Scientific, Waltham, MA, USA) and Alexa Fluor 647-conjugated secondary antibody (1:1000, Abcam) were used for fluorescence detection.

### 2.5. Western Blot Assay

Cells were collected and lysed in RIPA buffer (Thermo Fisher Scientific) containing a protease inhibitor cocktail (Solarbio, Beijing, China) for 20 min on ice. The lysates were clarified by centrifugation at 12,000× *g* for 20 min at 4 °C. The resulting supernatants were transferred to fresh tubes and combined with LDS sample buffer (GenScript), followed by heating at 95 °C for 5 min to denature the proteins. An equal quantity of total protein from each sample was resolved on 4–20% Sure PAGE gels (GenScript) at a constant voltage of 120 V. The separated proteins were subsequently transferred onto PVDF membranes (Merck Millipore, Boston, USA). Membranes were blocked with 5% bovine serum albumin (BSA, Sigma) for 2 h and incubated with primary antibodies at 4 °C overnight. The primary antibodies included anti- ARL13B (1:1000, Proteintech), anti-ACTUB (1:2000, Sigma), anti-DLG associated protein 5 (DLGAP5, 1:1000, Proteintech), anti-vasohibin 2 (VASH2, 1:1000, Proteintech), anti-kinesin family member 24 (KIF24, 1:500, Solarbio) and anti-GAPDH (1:5000, Proteintech). Following washing with TBST, HRP-conjugated secondary antibodies (1:4000, Proteintech) were applied for 2 h at room temperature. After additional TBST washes, immunoreactive bands were detected with an ECL chemiluminescence reagent (Aoqing Biotechnology), and images were obtained using a ChemiDoc MP Imaging System (Bio-Rad, Hercules, CA, USA).

### 2.6. RNA Sequencing and Transcriptomic Analysis

Transcriptomic profiling was performed by Novogene (Beijing, China) using total RNA isolated from serum-starved ARPE-19 cells in the presence or absence of kaempferol. Briefly, ARPE-19 cells were subjected to simultaneous serum reduction (0.5% FBS) and Kae (5 μM) treatment for a total duration of 24 h to induce ciliogenesis and evaluate transcriptomic responses. Cells maintained under the same conditions without kaempferol exposure served as the Blank control. RNA quantity was determined using a Nano-300 micro-spectrophotometer (Allsheng, Hangzhou China), and RNA integrity was assessed with an Agilent 2100 Bioanalyzer (Agilent Technologies, Santa Clara, CA, USA). The clustering of the index-coded samples was performed on a cBot Cluster Generation System using TruSeq PE Cluster Kit v3-cBot-HS (Illumina) according to the manufacturer’s instructions. After cluster generation, the library preparations were sequenced on an Illumina Novaseq platform and 150-bp paired-end reads were generated. Raw reads of FASTQ format were firstly processed through FASTQ software (v 0.23.4), and Gene expression levels were quantified based on read counts using feature counts v1.5.0-p3. Differentially expressed genes (DEGs) between groups were identified using the DESeq2 R package (1.20.0), with |log_2_ fold change| ≥ 1.0 and adjusted *p* value < 0.05 considered statistically significant. Gene Ontology (GO) and Kyoto Encyclopedia of Genes and Genomes (KEGG) pathway enrichment analyses were performed using cluster Profiler R, with adjusted *p* value < 0.05 indicating significant enrichment. Principal component analysis (PCA) and data visualization were conducted using R software (v 3.2.0).

### 2.7. Transmission Electron Microscopy (TEM)

Two-month-old male SPF C57BL/6 mice were utilized in accordance with protocols approved by the Experimental Animal Ethics Committee of the Institute of Basic Theory for Chinese Medicine, China Academy of Chinese Medical Sciences (Approval No.: IBTCMCACMS21-2507-01). Mice were anesthetized via intraperitoneal injection of tribromoethanol (0.2 mL/10 g body weight) and underwent surgical tracheal exposure. A dose of 25 μg SARS-CoV-2 NSP13 plasmid, complexed with in vivo-jetPEI (Polyplus-transfection, Illkirch, France), was delivered via intratracheal instillation; vehicle control mice received sterile saline. Subsequently, mice were orally administered kaempferol (12.5, 25, or 50 mg/kg) at 6, 24, and 48 h post-transfection. These dosage regimens correspond to 12.5, 25, and 50 mg/kg/day over two consecutive days, which were calculated using human-to-mouse body surface area conversion to represent clinically feasible oral intake levels of natural flavonols.

One hour after the final dose, harvested tracheal tissues were pre-fixed in 2.5% glutaraldehyde for 4 h, and replaced with 1% Osmium tetroxide for 1 h. Following dehydration through a graded ethanol series (30–100%), tissues were infiltrated with mixtures of ethanol and EPON-812 resin (3:1, 1:1, and 1:3, *v*/*v*) followed by pure resin, and polymerized at 65 °C for 48 h. Ultrathin sections (70–100 nm) were cut, mounted on Formvar/carbon-coated copper grids, double-counterstained with uranyl acetate (7 min at 4 °C) and lead citrate (3 min at room temperature), and observed using a Tecnai G2 Spirit BioTWIN transmission electron microscope (FEI Company, Hillsboro, OR, USA).

### 2.8. Statistical Analysis

Each experiment was conducted independently at least three times. Quantitative data are expressed as the mean ± SEM. Statistical comparisons between two groups were performed using a two-tailed unpaired Student’s *t*-test, whereas datasets involving three or more groups were analyzed by one-way analysis of variance (ANOVA). Statistical analyses were conducted using GraphPad Prism 8.0, and *p* < 0.05 was considered statistically significant.

## 3. Results

### 3.1. Kae Regulates the Microtubule-Associated Pathways Related to Ciliogenesis

Kae is a naturally occurring flavonoid widely distributed in medicinal plants and dietary vegetables [12]. Previous studies have shown that Kae regulates cytoskeletal organization by modulating cytoskeletal components and microtubule stability [15]. As cilia are highly specialized microtubule-based organelles that require cytoskeletal homeostasis for assembly and maintenance [16], we hypothesized that Kae may regulate ciliogenesis through cytoskeleton-associated pathways. To comprehensively identify the molecular events, transcriptomic profiling was performed in serum-starved ARPE-19 cells treated with Kae.

**Figure 1:**
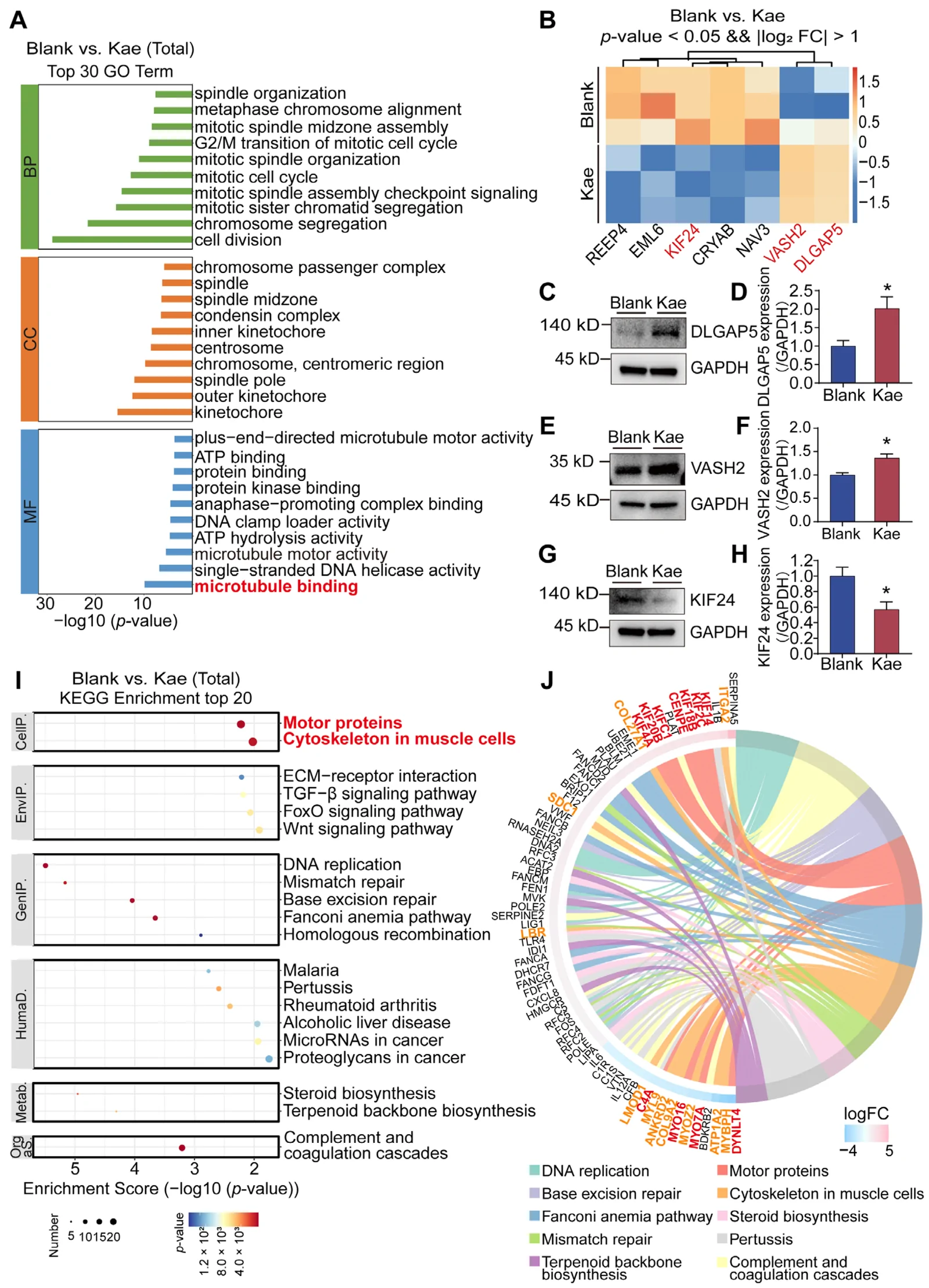
Transcriptomic analysis reveals Kae-induced alterations in cytoskeleton and motor protein pathways. (**A**) Functional enrichment analysis of differentially expressed genes (DEGs) using Gene Ontology (GO) between the Kae-treated and Blank control group under starvation conditions. The top 30 significantly enriched GO terms are categorized into Biological Process (BP), Cellular Component (CC), and Molecular Function (MF). (**B**) Hierarchical clustering heatmap of selected representative DEGs between the Blank and Kae group (|log_2_ FC| > 1 with *p* < 0.05). Red and blue denote up-regulation and down-regulation, respectively. (**C**,**D**) Representative Western blot image showing the expression level of DLGAP5 after 5 μM Kae treatment and the densitometric quantification of normalized DLGAP5 protein level, with GAPDH as the loading control. (**E**,**F**) Representative Western blot image showing the expression level of VASH2 after 5 μM Kae treatment and the densitometric quantification of normalized VASH2 protein level. (**G**,**H**) Representative Western blot image showing the expression level of KIF24 after 5 μM Kae treatment and the densitometric quantification of normalized KIF24 protein level. Data are presented as mean ± SEM (*n* = 3). * *p* < 0.05 vs. Blank. (**I**) Pathway enrichment analysis based on the Kyoto Encyclopedia of Genes and Genomes (KEGG) showing the top 20 enriched pathways for Kae vs. Blank group. Enriched pathways are classified into functional categories, highlighting key pathways related to the motor proteins and the cytoskeleton. (**J**) Chord plot illustrating the relationships between key DEGs and their associated enriched KEGG pathways along with their corresponding log_2_ fold change (log FC) values.

Gene Ontology (GO) enrichment analysis revealed that the Kae-responsive differentially expressed genes (DEGs) were highly enriched in terms that were directly associated with microtubule dynamic organization and chromosome behavior in the biological processes (BP), cellular component (CC), and molecular function (MF) categories (Figure 1A). Key enriched processes included spindle organization, chromosome segregation, centrosome, and importantly microtubule binding and plus-end-directed microtubule motor activity, implying that Kae generally modulates microtubule-associated mechanical activities and structural remodeling (Figure 1A). A hierarchical clustering heatmap analysis also identified a subset of critical genes that control microtubule dynamics and ciliary assembly (Figure 1B). In particular, Kae significantly upregulated microtubule-associated disks large-associated protein 5 (*DLGAP5*) that promotes microtubule polymerization and spindle stabilization [17], and vasohibin 2 (*VASH2*), which is a regulator of tubulin post-translational modification and microtubule dynamics [18]. On the other hand, Kae strongly down-regulated kinesin family member 24 (*KIF24*), a kinesin located at the centrosome, which has been shown to actively depolymerize centriolar microtubules and inhibit ciliary assembly [19]. Moreover, important regulators of cytoskeleton and membrane organization, including neuron navigator 3 (*NAV3*) [20], crystallin alpha B (*CRYAB*) [21,22], EMAP like 6 (*EML6*) [23] and receptor accessory protein 4 (*REEP4*) were also down-regulated [24], suggesting that together, Kae induces broad remodeling of cytoskeletal and microtubule-associated regulatory networks. To further validate these transcriptomic changes at the protein level, we selected three representative DEGs closely associated with microtubule dynamics and ciliary regulation, namely DLGAP5, VASH2, and KIF24, for Western blot analysis. Consistent with the RNA-seq results, Kae treatment significantly increased the protein levels of DLGAP5 and VASH2, while markedly decreasing KIF24 expression (Figure 1C–H). The concordance between the transcriptomic and protein-level changes further supports the involvement of microtubule-associated regulatory pathways in the cellular response to Kae.

To further understand the signaling network involved, we performed Kyoto Encyclopedia of Genes and Genomes (KEGG) analysis on major biological categories (Figure 1I). Intriguingly, the DEGs in cellular processes were enriched mainly in motor proteins and cytoskeleton in muscle cells (Figure 1I). Detailed functional network analysis through chord plot confirmed specific DEGs converging onto these structural pathways (Figure 1J). In the motor protein pathway, Kae significantly regulated several kinesin family members (*KIF14*/*2C*/*18B*/*C1*/*20B*/*4A*) [25,26,27], centromere protein E (*CENPE*) [28], myosin 16/7A (*MYO16*/*7A*) [29], and dynein light chain tctex-type 4 (*DYNLT4*) [30], suggesting broad modulation of microtubule- and actin-dependent intracellular transport mechanisms. Similarly, DEGs enriched in the Cytoskeleton in muscle cells pathway included core machinery regulating extracellular matrix anchoring, such as integrin subunit alpha 2 (*ITGA2*) [31], collagen type XXVII alpha 1 chain (*COL27A1*) [32,33], collagen type IX alpha 2 chain (*COL9A2*) [34], and syndecan 1 (*SDC1*) [35,36], actomyosin contractility like leiomodin 1 (*LMOD1*) [37], myosin light chain 9 (*MYL9*) [38], myozenin 2 (*MYOZ2*) [39], myosin binding protein H (*MYBPH*) [40], and ankyrin repeat domain 2 (*ANKRD2*) [41], and membrane-cytoskeleton integrity including lamin B receptor (*LBR*) [42] and ATPase Na^+^/K^+^ transporting subunit alpha 2 (*ATP1A2*) [43], indicating a general reorganization of structural homeostasis.

Taken together, transcriptomic analysis revealed that Kae treatment is associated with extensive remodeling of microtubule- and cytoskeleton-related gene networks, including regulators of microtubule dynamics and intracellular motor activity. Given that cilia are microtubule-based organelles whose axonemal microtubules provide the structural scaffold for ciliary assembly and maintenance, alterations in microtubule dynamics and stability may influence ciliary morphology and length [44,45]. These transcriptomic and protein level alterations therefore provided a rationale for further investigating whether modulation of microtubule stability contributes to the cilia promoting effect of Kae.

### 3.2. Kae Promotes Ciliary Elongation Without Affecting Ciliogenesis Initiation

**Figure 2:**
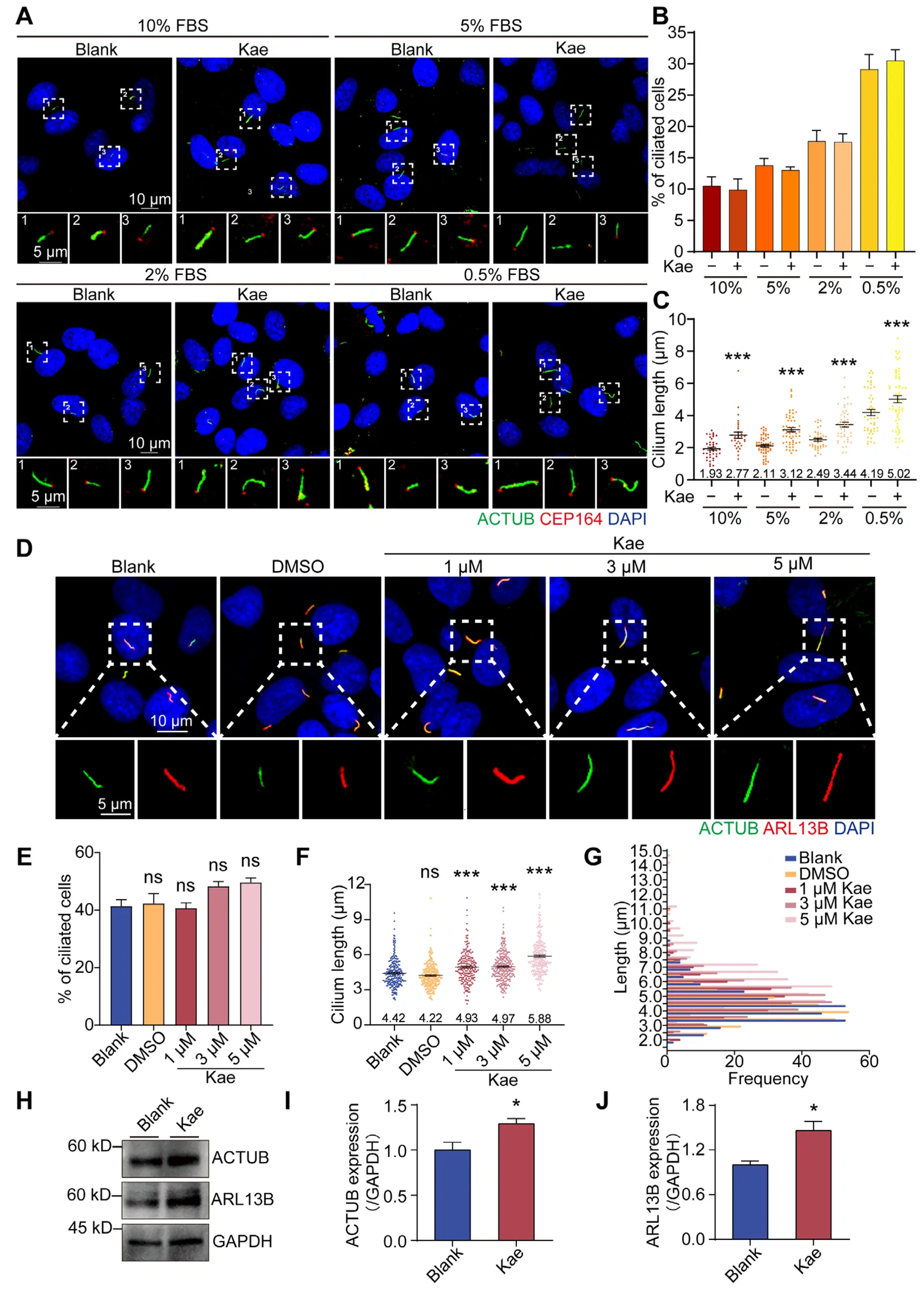
Kae regulates cilia length in a concentration-dependent manner. (**A**) Representative IF images of cilia in ARPE-19 cells treated with Kae for 24 h under different serum concentrations (10%, 5%, 2%, and 0.5%). Cilia were stained with CEP164 (red) and ACTUB (green); nuclei were stained with DAPI (blue). Scale bars: 10 μm (upper panels) and 5 μm (lower panels). (**B**,**C**) Quantitative analysis of cilia characteristics, including ciliation frequency (**B**) and ciliary length (**C**). Data are presented as mean ± SEM (Cilia length was measured across 10 fields of view, with approximately 10–20 cilia measured per field of view); *** *p* < 0.005 vs. Blank. (**D**) Representative IF images of cilia. ARPE-19 cells were treated with DMSO or increasing concentrations of Kae (1 μM, 3 μM, and 5 μM) under serum-starved conditions to induce ciliogenesis. Cilia were stained with ARL13B (red) and ACTUB (green); nuclei were stained with DAPI (blue). Scale bars: 10 μm (upper panels) and 5 μm (lower panels). (**E**–**G**) Quantitative analysis of cilia characteristics, including ciliation frequency (**E**), ciliary length (**F**), and length frequency distribution (**G**). Data are presented as mean ± SEM (Cilia length was measured across 10 fields of view, with approximately 40–50 cilia measured per field of view); ns, not significant; *** *p* < 0.005 vs. DMSO. (**H**) Representative Western blot images showing the expression levels of cilia marker ACTUB and ARL13B after 5 μM Kae treatment, with GAPDH as the loading control. (**I**,**J**) Densitometric quantification of the normalized ACTUB (**I**) and ARL13B (**J**) protein levels. Data are presented as mean ± SEM (*n* = 3). * *p* < 0.05 vs. Blank.

To determine whether the transcriptomic alterations associated with microtubule dynamics were accompanied by functional changes in cilia, we first evaluated the effects of Kae on cilium formation and length under different serum conditions, including conventional serum-containing and serum-starved conditions. Because serum deprivation is commonly used to induce ciliogenesis but may itself impose cellular stress and alter cytoskeletal dynamics, we sought to determine whether the effects of Kae on ciliary length were dependent on serum starvation [46]. ARPE-19 cells were cultured in medium containing 10%, 5%, 2%, or 0.5% serum and treated with Kae. Because cilium length can undergo rapid dynamic regulation, with changes detectable within 3 h, independent of changes in the proportion of ciliated cells, we first examined the early 3-h response [47]. Ciliation frequency was lower under higher serum conditions (10%, 5%, and 2%) than under 0.5% serum. Notably, Kae increased ciliary length across the tested serum concentrations without a corresponding increase in ciliation frequency (Figure S1A–C). We then focused on the 24-h treatment condition to characterize the effects of Kae on cilia in greater detail. At 24 h, immunofluorescence analysis showed that the proportion of ciliated cells was relatively low under higher serum conditions (10%, 5%, and 2%), whereas 0.5% serum supported a higher frequency of cilia formation. Despite these differences in ciliation frequency, Kae treatment consistently increased cilium length across the tested serum concentrations, without a corresponding increase in the proportion of ciliated cells (Figure 2A–C). The proportion of ciliated cells remained relatively low under higher serum conditions and was higher under 0.5% serum. Specifically, the ciliation frequency was 10.50 ± 1.41%, 13.75 ± 1.13%, 17.63 ± 1.72%, and 29.13 ± 2.36% under 10%, 5%, 2%, and 0.5% serum, respectively, in the control groups, compared with 9.88 ± 1.75%, 13.00 ± 0.53%, 17.50 ± 1.34%, and 30.50 ± 1.76%, respectively, in the Kae-treated groups (Figure 2B). In contrast, the mean cilium length under 10%, 5%, 2%, and 0.5% serum was 1.93 ± 0.10 μm, 2.11 ± 0.08 μm, 2.49 ± 0.10 μm, and 4.19 ± 0.19 μm, respectively, in the control groups, whereas Kae treatment increased these values to 2.77 ± 0.18 μm, 3.12 ± 0.13 μm, 3.44 ± 0.15 μm, and 5.02 ± 0.22 μm, respectively (Figure 2C). These findings suggest that Kae primarily promotes ciliary elongation rather than ciliogenesis, with this effect detectable at an early stage of ciliary formation and maintained across different serum conditions, indicating that Kae-mediated ciliary elongation is not restricted to serum-starved conditions.

Based on these findings and the established use of low-serum culture to promote ciliogenesis, 0.5% serum was selected for subsequent experiments because it supported a relatively higher ciliation frequency while preserving a robust elongation response to Kae. We next evaluated the effects of different concentrations of Kae on ciliary phenotypes. To minimize potential cytotoxicity at higher concentrations, relatively low concentrations of Kae (1 μM, 3 μM, and 5 μM) were selected. ARPE-19 cells were serum-starved to induce ciliogenesis and treated with Kae for 24 h, alongside Blank and vehicle (DMSO) controls. Immunofluorescence co-staining for acetylated α-tubulin (ACTUB, green) identifying the stabilized axonemal microtubule backbone, and ADP-ribosylation factor-like protein 13B (ARL13B, red) to label the ciliary membrane demonstrated distinct cilia across all experimental conditions (Figure 2D). Quantitative assessment showed that Kae treatment did not alter the proportion of ciliated cells relative to the control groups, maintaining a ciliation frequency of about 40–50% across all conditions (Figure 2E). In contrast, Kae induced a pronounced, concentration-dependent increase in cilium length (Figure 2F). While the mean cilium length was 4.22 ± 0.07 μm in the DMSO control group, Kae significantly extended ciliary length to 4.93 ± 0.09 μm, 4.97 ± 0.07 μm, and 5.88 ± 0.09 μm following treatment with 1 μM, 3 μM, and 5 μM Kae, respectively. Frequency distribution analysis confirmed a rightward shift toward longer ciliary populations following Kae treatment, particularly at 5 μM (Figure 2G). To further determine whether the ciliary elongation effect of Kae was reproducible across different cell types, we subsequently examined its effects in MRC-5 and RPE-1 cells under the same 0.5% serum conditions. Consistent with the findings in ARPE-19 cells, treatment with Kae at 1 μM, 3 μM, and 5 μM significantly increased cilium length in both MRC-5 and RPE-1 cells, without a corresponding increase in the proportion of ciliated cells (Figure S2A–H). These findings further support the reproducibility of the ciliary elongation effect of Kae in multiple cell models, without evidence for a corresponding increase in ciliation frequency.

To assess whether this morphological elongation correlates with ciliary protein stabilization, we examined the expression of axonemal and ciliary membrane markers by Western blotting. Compared with the control group, Kae treatment consistently increased the protein levels of both ACTUB and ARL13B (Figure 2H–J). Together, these findings indicate that Kae selectively drives cilium elongation rather than initiating ciliary assembly, which is accompanied by the upregulation of axonemal and membrane-associated ciliary components.

### 3.3. Kae Rescues NSP13-Induced Ciliary Defects

Having established that Kae promotes ciliary elongation under physiological conditions, we next investigated whether Kae exerts a protective effect against pathological ciliary injury. SARS-CoV-2 nonstructural protein 13 (NSP13), a highly conserved viral helicase, interacts with centrosomal components and disrupts host organelle homeostasis [48]. We therefore evaluated whether NSP13 induces ciliary injury and whether Kae treatment could restore these defects.

**Figure 3:**
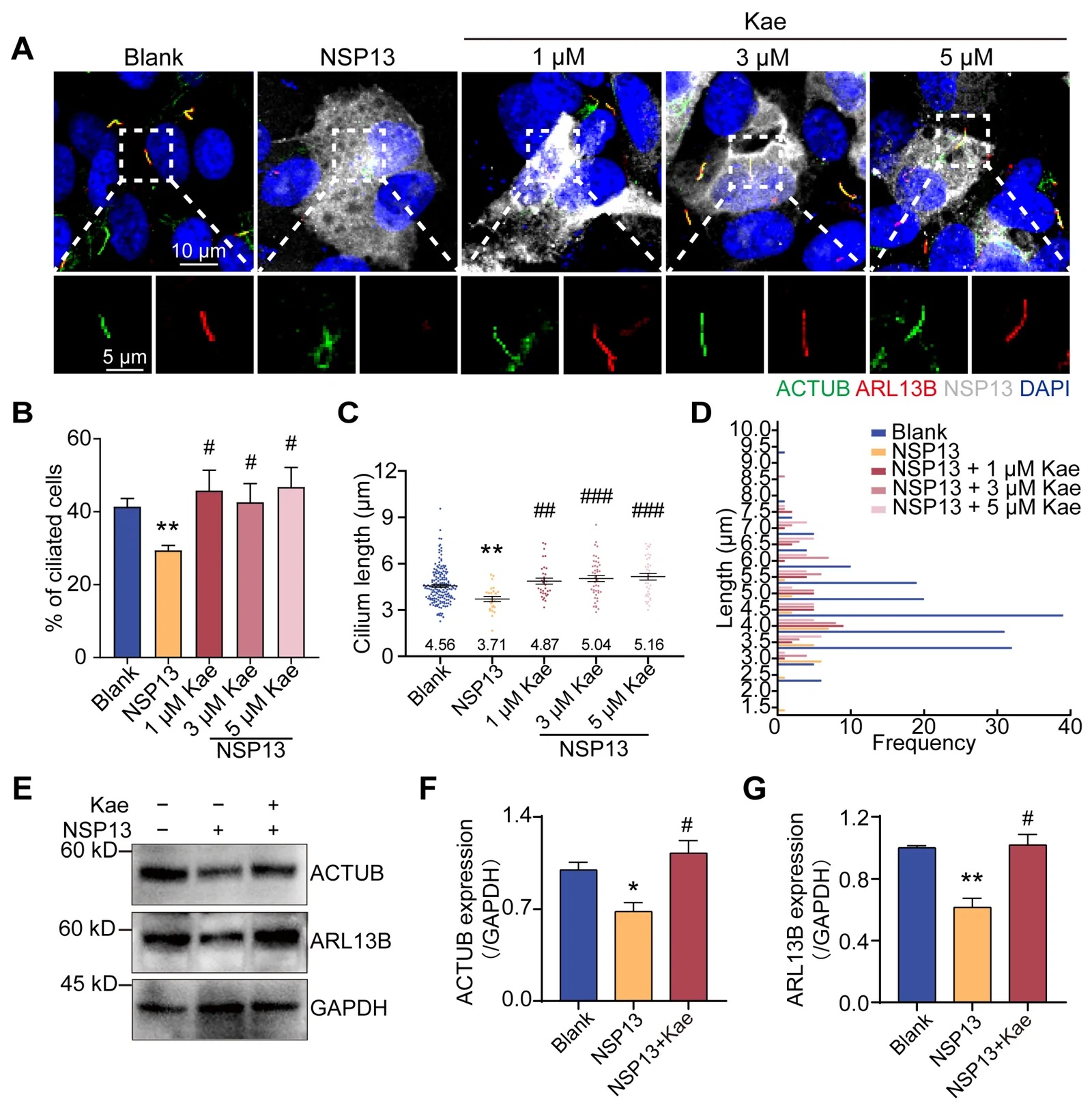
Kae rescues NSP13-induced cilia defects. (**A**) Representative IF images of cilia. ARPE-19 cells were transfected with NSP13 (gray) and subsequently treated with increasing concentrations of Kae (1 μM, 3 μM, and 5 μM) under serum-starved conditions to induce ciliogenesis. Cilia were stained with ARL13B (red), ACTUB (green), and NSP13 (gray); nuclei were stained with DAPI (blue). Scale bars: 10 μm (upper panels) and 5 μm (lower panels). (**B**–**D**) Quantitative analysis of cilia characteristics in Kae-rescued NSP13-transfected cells, including ciliation frequency (**B**), ciliary length (**C**), and length frequency distribution (**D**). Data are presented as mean ± SEM (Cilia length was measured across 10 fields of view, with approximately 10–20 cilia measured per field of view); ** *p* < 0.01 vs. Blank. # *p* < 0.05, ## *p* < 0.01, ### *p* < 0.005 vs. NSP13. (**E**) Representative Western blot images showing ACTUB and ARL13B protein levels in NSP13-transfected cells treated with Kae, with GAPDH used as the loading control. (**F**,**G**) Densitometric quantification of normalized ACTUB (**F**) and ARL13B (**G**) protein levels. Data are presented as mean ± SEM (*n* = 3); * *p* < 0.05, ** *p* < 0.01 vs. Blank. # *p* < 0.05 vs. NSP13.

Serum-starved ARPE-19 cells expressing NSP13 were treated with vehicle or increasing concentrations of Kae (1 μM, 3 μM, and 5 μM) for 24 h. Immunofluorescence staining was performed for ACTUB (green, axoneme), ARL13B (red, ciliary membrane), and NSP13 (gray, transfected cells) (Figure 3A). Compared with the Blank group, NSP13 expression markedly disrupted ciliary integrity, characterized by marked loss of ARL13B-positive cilia and shortening or fragmentation of the residual ACTUB-positive axonemal structures (Figure 3A). As noted, the apparent loss of ARL13B fluorescence reflects a profound pathological phenotype in which NSP13 preferentially targets ciliary membrane integrity, leading to the selective loss or mislocalization of membrane-associated proteins. Quantitative analysis revealed that NSP13 expression significantly reduced the percentage of ciliated cells from 41.40 ± 2.25% in the Blank group to 29.40 ± 1.36% (Figure 3B). Remarkably, Kae treatment effectively rescued this deficit, restoring the ciliation frequency to 43.20 ± 7.97%, 42.60 ± 5.11%, and 46.80 ± 5.32% at concentrations of 1 μM, 3 μM, and 5 μM Kae, respectively (Figure 3B).

Similarly, NSP13 expression caused a sharp reduction in average cilium length from 4.56 ± 0.09 μm in the Blank group to 3.71 ± 0.17 μm (Figure 3C). Kae treatment concentration-dependently reversed this ciliary shortening, extending the mean cilium length to 4.87 ± 0.20 μm, 5.04 ± 0.19 μm, and 5.16 ± 0.21 μm after treatment with 1 μM, 3 μM, and 5 μM Kae, respectively (Figure 3C). Frequency distribution analysis further verified a distinct shift from a shorter ciliary population in NSP13-expressing cells toward longer ciliary profiles following Kae co-treatment (Figure 3D).

To confirm these morphological rescues at the molecular level, we assessed the expression of ciliary structural proteins by Western blotting (Figure 3E–G). Compared with the Blank control, NSP13 expression significantly suppressed the protein levels of both ACTUB and ARL13B. Co-treatment with 5 μM Kae completely restored the expression of ACTUB and ARL13B compared with the NSP13 group, indicating restoration of ciliary structural components.

Collectively, these findings provide evidence that SARS-CoV-2 NSP13 causes severe ciliary disassembly and shortening, whereas Kae effectively protects against NSP13-induced ciliary pathology by restoring axonemal and membrane structural protein expression.

### 3.4. Kae Restores Ciliary Length Following Ciliobrevin D-Induced Dynein Inhibition

**Figure 4:**
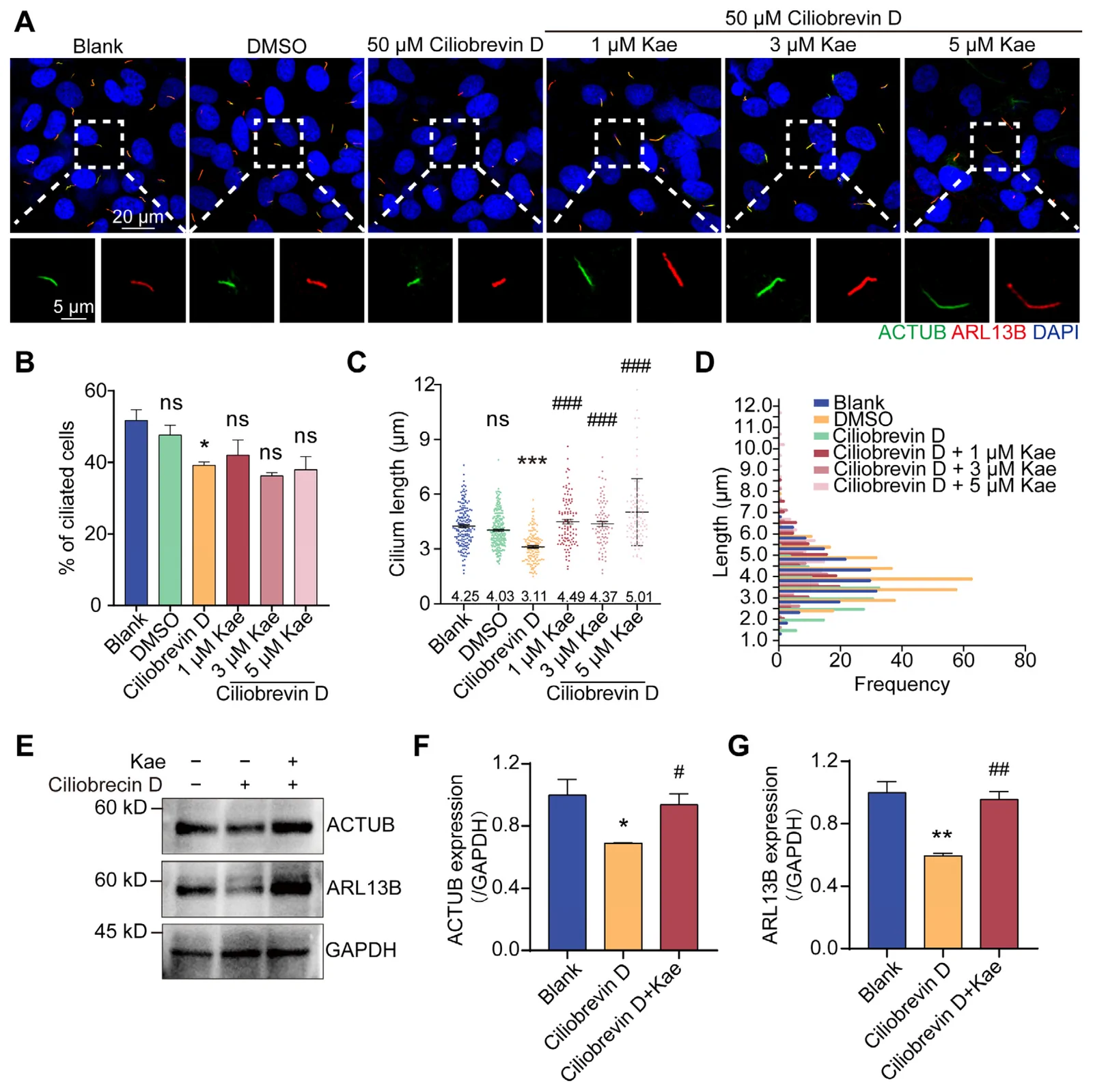
Kae restores ciliobrevin D-induced cilia shortening. (**A**) Representative IF images of cilia. ARPE-19 cells were treated with ciliobrevin D (50 µM) and subsequently exposed to increasing concentrations of Kae (1 μM, 3 μM, and 5 μM) under serum-starved conditions to induce ciliogenesis. Cilia were stained with ARL13B (red) and ACTUB (green); nuclei were stained with DAPI (blue). Scale bars: 20 μm (upper panels) and 5 μm (lower panels). (**B**–**D**) Quantitative analysis of cilia characteristics in ciliobrevin D-treated cells following Kae treatment, including ciliation frequency (**B**), ciliary length (**C**), and length frequency distribution (**D**). Data are presented as mean ± SEM (Cilia length was measured across 10 fields of view, with approximately 20–30 cilia measured per field of view); ns, not significant; * *p* < 0.05, *** *p* < 0.005 vs. DMSO. ### *p* < 0.005 vs. Ciliobrevin D. (**E**) Representative Western blot images of ACTUB and ARL13B in cells treated with ciliobrevin D and Kae, with GAPDH used as the loading control. (**F**,**G**) Densitometric quantification of ACTUB (**F**) and ARL13B (**G**) protein levels. Data are presented as mean ± SEM (*n* = 3); * *p* < 0.05, ** *p* < 0.01 vs. Blank. # *p* < 0.05, ## *p* < 0.01 vs. Ciliobrevin D.

To determine whether the protective effect of Kae extends beyond viral-associated ciliary injury to a distinct form of ciliary dysfunction involving dynein-dependent transport, we established a pharmacological model using Ciliobrevin D, a small-molecule inhibitor of cytoplasmic dynein, the motor responsible for retrograde transport along microtubules [49]. Because dynein-mediated retrograde transport is closely linked to IFT, inhibition of dynein can perturb ciliary transport and consequently affect ciliary homeostasis [26]. Serum-starved ARPE-19 cells were treated with 50 μM Ciliobrevin D in the absence or presence of increasing concentrations of Kae (1 μM, 3 μM, and 5 μM) for 24 h, followed by immunofluorescence staining with ARL13B (red) and ACTUB (green) to visualize cilia architecture (Figure 4A).

Quantitative analysis revealed that Ciliobrevin D treatment markedly reduced the proportion of ciliated cells from 47.68 ± 2.77% in the DMSO group to 39.20 ± 0.86% (Figure 4B). Co-treatment with Kae at 1 μM, 3 μM, or 5 μM did not significantly alter the ciliation percentage compared with Ciliobrevin D (42.00 ± 4.14%, 36.20 ± 1.02%, and 37.80 ± 3.60%, respectively), indicating that Kae does not rescue Ciliobrevin D-induced ciliary disassembly or loss.

In sharp contrast, Kae treatment effectively rescued Ciliobrevin D-induced ciliary shortening in a concentration-dependent manner (Figure 4C). While Ciliobrevin D reduced average cilium length from 4.03 ± 0.06 μm in the DMSO group to 3.11 ± 0.07 μm, addition of Kae markedly extended ciliary length to 4.49 ± 0.13 μm, 4.37 ± 0.15 μm, and 5.01 ± 0.19 μm with 1 μM, 3 μM, and 5 μM Kae, respectively (Figure 4C). Consistent with these findings, frequency distribution analysis confirmed that Ciliobrevin D shifted the ciliary length population toward shorter ranges, whereas Kae co-treatment progressively restored the frequency of longer cilia, particularly at 5 μM (Figure 4D).

To further examine whether Ciliobrevin D-induced ciliary shortening was accompanied by alterations in IFT organization, we analyzed the localization of IFT81, a core component of the IFT-B complex, together with the ciliary membrane marker ARL13B as a Supplementary Analysis (Figure S3A,B). In control cells, IFT81 showed a clear spatial association with ARL13B-positive cilia. Following Ciliobrevin D treatment, IFT81 remained detectable within the residual ARL13B-positive cilia and exhibited a relatively continuous distribution along the ciliary axis, without an obvious abnormal accumulation or redistribution. Notably, Kae treatment restored ciliary length while IFT81 localization remained detectable and associated with the elongated ARL13B-positive cilia. These findings suggest that, under our experimental conditions, Ciliobrevin D-induced ciliary shortening was not accompanied by an apparent disruption of IFT81 localization, whereas Kae-mediated ciliary elongation occurred with preservation of IFT81 localization within the cilium.

To further evaluate the effect of Kae on ciliary structural proteins, the expression levels of ACTUB and ARL13B were examined by Western blotting. Compared with the Blank group, Ciliobrevin D treatment significantly decreased the expression of both ACTUB and ARL13B (Figure 4E–G). In contrast, treatment with 5 μM Kae markedly restored the expression of these ciliary markers, consistent with the restoration of ciliary length and supporting a protective effect of Kae on ciliary structural integrity under dynein inhibition.

Collectively, these results demonstrate that Kae promotes the elongation of residual cilia following Ciliobrevin D-induced dynein inhibition and is associated with restoration of ACTUB and ARL13B expression. Together with the preservation of IFT81 localization, these findings support an association between Kae-mediated ciliary elongation and the maintenance of the axonemal microtubule-IFT environment.

### 3.5. Kae Rescues Cililary Length and Structural Protein in CEP290 Deficiency-Induced Ciliary Defects

**Figure 5:**
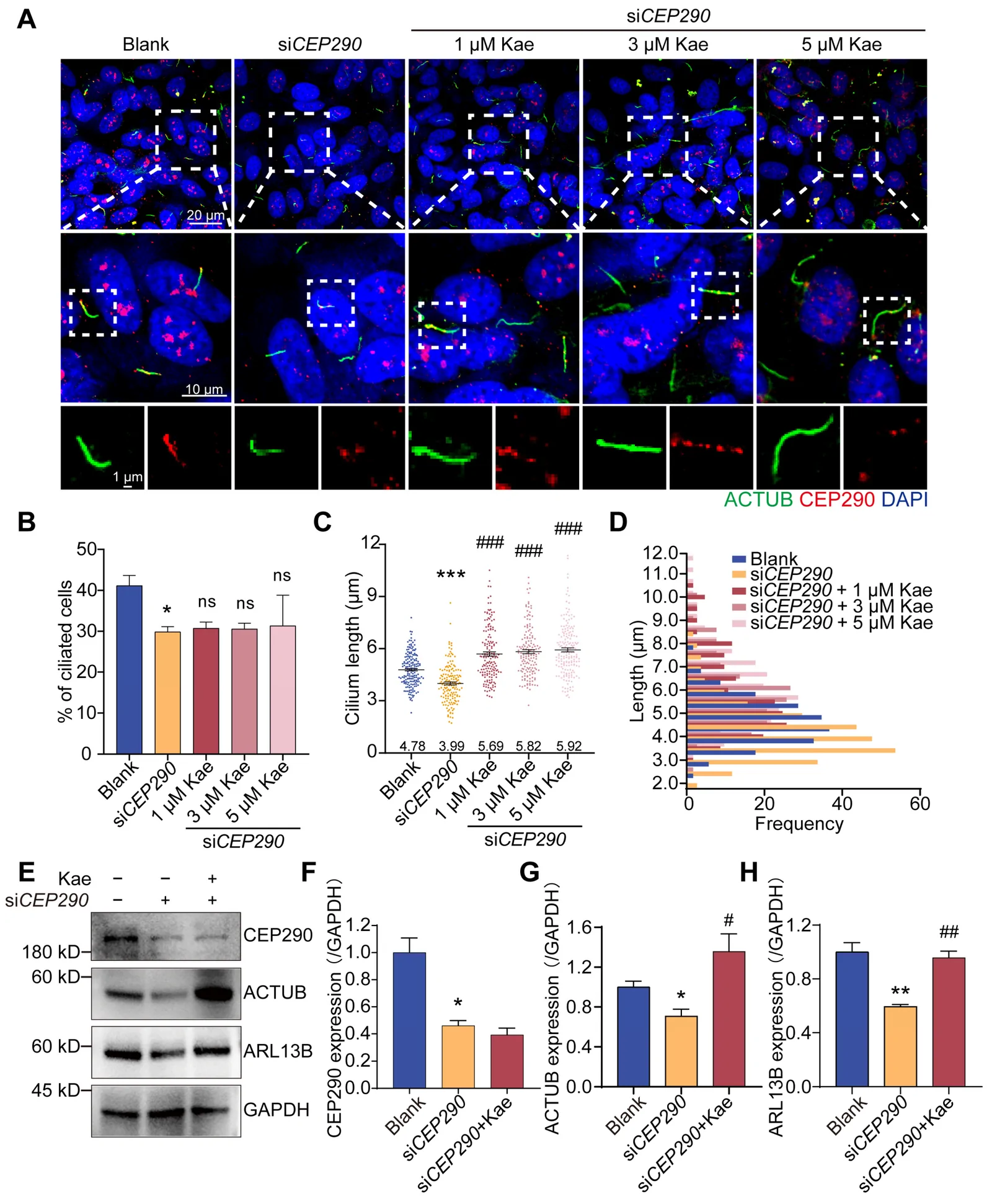
Kae restores cilia length in CEP290-deficient cells. (**A**) Representative IF images of cilia. ARPE-19 cells were transfected with control or si*CEP290*, and subsequently treated with increasing concentrations of Kae (1 μM, 3 μM, and 5 μM) under serum-starved conditions to induce ciliogenesis. Cilia were stained with ACTUB (green), CEP290 (red), and nuclei were stained with DAPI (blue). Scale bars: 20 μm (upper panels), 10 μm (middle panels) and 1 μm (lower panels). (**B**–**D**) Quantitative analysis of cilia characteristics in si*CEP290*-transfected cells treated with Kae, including ciliation frequency (**B**), ciliary length (**C**), and length frequency distribution (**D**). Data are presented as mean ± SEM (Cilia length was measured across 10 fields of view, with approximately 30–40 cilia measured per field of view); ns, not significant; * *p* < 0.05, *** *p* < 0.005 vs. Blank. ### *p* < 0.005 vs. si*CEP290*. (**E**) Representative Western blot images of ACTUB and ARL13B in cells transfected with si*CEP290* and treated with Kae, with GAPDH used as the loading control. (**F**–**H**) Densitometric quantification of CEP290 (**F**), ACTUB (**G**) and ARL13B (**H**) protein levels. Data are presented as mean ± SEM (*n* = 3); * *p* < 0.05, ** *p* < 0.01 vs. Blank. # *p* < 0.05, ## *p* < 0.01 vs. si*CEP290*.

To further determine whether Kae can rescue ciliary defects caused by dysfunction of early ciliogenesis and transition zone machinery, we established a basal body/transition zone injury model via CEP290 depletion. CEP290 plays a vital role in ciliary gate assembly, basal body docking, and cilium formation [50]. Serum-starved ARPE-19 cells were transfected with si*CEP290* and subsequently treated with vehicle or graded concentrations of Kae (1 μM, 3 μM, and 5 μM) for 24 h. Cilia and CEP290 localization were assessed by immunofluorescence staining for ACTUB (green) and CEP290 (red) (Figure 5A). Compared with the control group, CEP290 knockdown markedly reduced the frequency of ciliated cells and shortened the length of the remaining cilia. Kae treatment significantly increased the length of the surviving cilia in a concentration-dependent manner (Figure 5A). Notably, CEP290 fluorescence remained detectable within the ciliary compartment after Kae treatment, with an apparently more continuous signal along the residual ciliary structures. This spatial pattern should be interpreted cautiously, as it may reflect the presence and redistribution of residual CEP290 following incomplete knockdown rather than restoration of normal CEP290 expression or transition-zone architecture. Together, these findings indicate that Kae promotes the elongation of surviving cilia following CEP290 depletion without restoring ciliation frequency or overall CEP290 abundance.

Quantitative analysis demonstrated that CEP290 knockdown significantly compromised ciliogenesis, reducing the percentage of ciliated cells from 41.12 ± 2.51% in control cells to 29.84 ± 1.29% (Figure 5B). Co-treatment with Kae at 1, 3, or 5 μM did not markedly change the proportion of ciliated cells relative to the si*CEP290* exposure (30.73 ± 1.49%, 30.53 ± 1.44%, and 31.33 ± 3.35%, respectively), indicating that Kae did not primarily rescue the initiation of cilium formation.

In contrast, Kae application markedly rescued CEP290 deficiency-induced ciliary shortening in a concentration-dependent manner (Figure 5C). While si*CEP290* reduced the average cilium length from 4.78 ± 0.07 μm (Blank) to 3.99 ± 0.09 μm (si*CEP290*), supplementation of Kae extended the mean cilium length to 5.69 ± 0.12 μm, 5.82 ± 0.11 μm, and 5.92 ± 0.11 μm, respectively. Notably, Kae treatment restored cilium length beyond the basal levels observed in Blank control cells. Frequency distribution analysis further verified that CEP290 knockdown shifted the ciliary length distribution toward shorter ranges, whereas Kae addition progressively increased the proportion of longer cilia across all tested doses (Figure 5D).

To validate these observations at the protein level, we first confirmed the knockdown efficiency of CEP290 and then examined the expression of CEP290, as well as the ciliary markers ACTUB and ARL13B, by Western blotting (Figure 5E–H). Compared with the Blank group, si*CEP290* markedly reduced the overall protein level of CEP290, confirming the efficacy of the knockdown. Notably, co-treatment with 5 μM Kae did not restore or increase the overall cellular protein abundance of CEP290 in si*CEP290*-transfected cells. In contrast, knockdown of CEP290 significantly reduced the protein levels of both ACTUB and ARL13B compared with the Blank group, whereas co-treatment with 5 μM Kae significantly upregulated the expression of these ciliary-associated proteins. These findings indicate that Kae can restore ciliary-associated protein levels in the context of CEP290 depletion without restoring the overall cellular abundance of CEP290, suggesting that the Kae-mediated rescue of ciliary elongation may not depend on restoration of CEP290 protein expression but may instead be associated with the stabilization of axonemal microtubules.

Collectively, these findings demonstrate that Kae effectively alleviates CEP290-associated ciliary defects by restoring axonemal microtubule stability, although it does not fully rescue the impairment in ciliogenesis initiation or ciliated cell frequency.

### 3.6. Kaempferol Promotes Microtubule Recovery and Preserves Ciliary Ultrastructure

**Figure 6:**
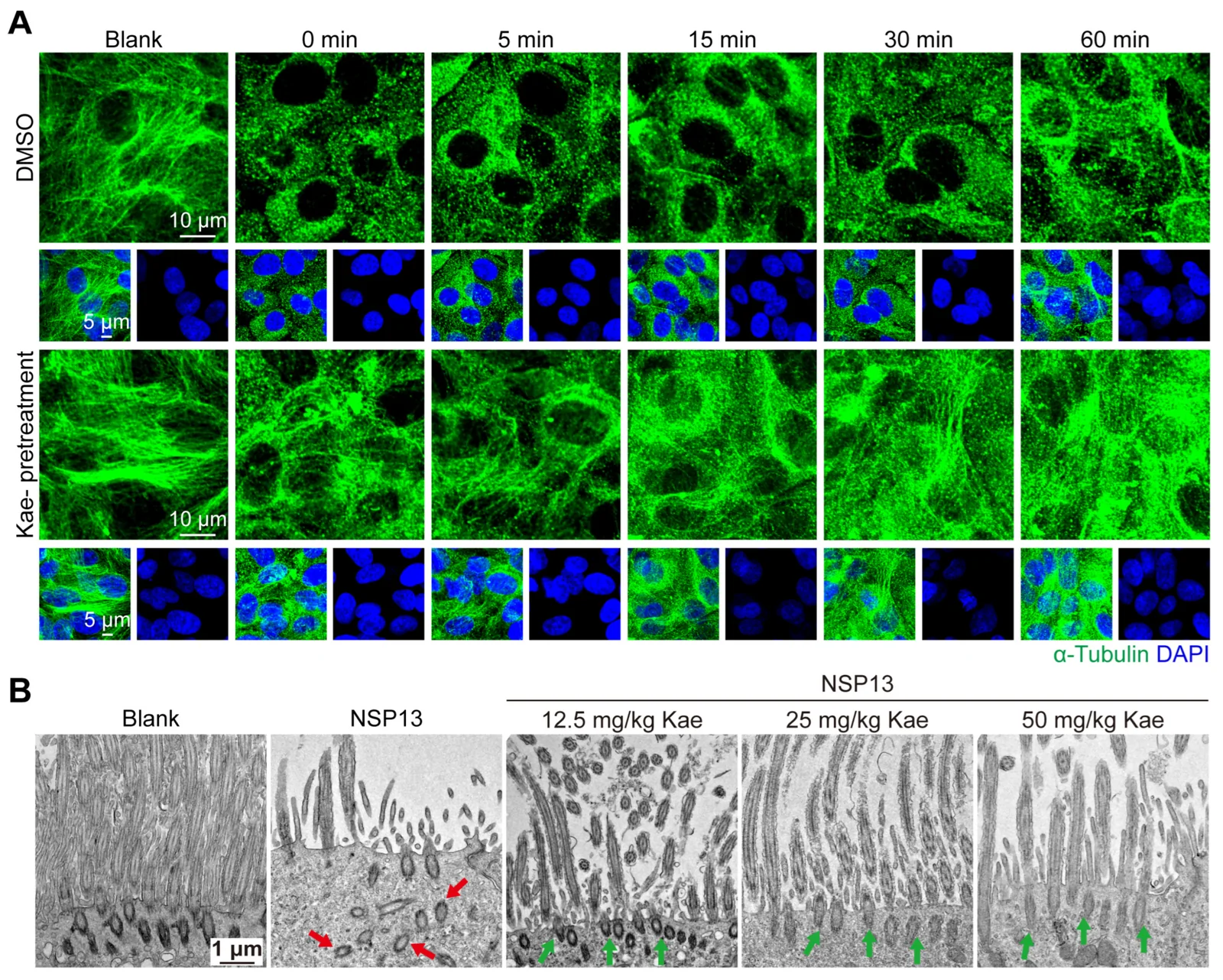
Kae restores cytoskeletal architecture in vitro and ciliary ultrastructure in vivo. (**A**) ARPE-19 cells pretreated with DMSO or KAE (5 μM) for 24 h were exposed to nocodazole (10 μM) for 30 min, then washed and allowed to recover for the indicated times (0, 5, 15, 30, and 60 min). Representative IF images of α-tubulin (green) and DAPI (blue) are shown. Scale bars: 10 μm (upper panels) and 5 μm (lower panels). (**B**) Representative transmission electron microscopy (TEM) images of tracheal epithelial cilia from Blank, NSP13-transfected, and NSP13-transfected mice treated with Kae (*n* = 3 mice per group). Scale bars: 1 μm. Red arrows indicate abnormally localized basal bodies, whereas green arrows indicate normally localized basal bodies.

To further investigate whether microtubule stabilization contributes to the cilia-elongating activity of Kae, we examined microtubule dynamics following nocodazole-induced depolymerization. Because the ciliary axoneme is composed of highly stable microtubule doublets and its length is tightly regulated by the balance between microtubule assembly and disassembly, alterations in microtubule dynamics can influence ciliary structure and length [44]. ARPE-19 cells were pretreated with DMSO or Kae (5 μM) for 24 h, followed by nocodazole treatment to induce microtubule depolymerization. Nocodazole markedly reduced intact α-Tubulin-positive microtubule fibers in control cells, which appeared predominantly as diffuse punctate or fragmented signals. In contrast, relatively continuous microtubule fibers remained detectable in Kae-pretreated cells following nocodazole exposure. After nocodazole washout, microtubule networks progressively reassembled in both groups, whereas Kae-pretreated cells displayed more prominent restoration of continuous and robust microtubule fibers throughout the recovery period (Figure 6A). These results indicate that Kae increases the resistance of the microtubule network to depolymerization and facilitates microtubule recovery.

We next asked whether the stabilizing effects of Kae on microtubule dynamics translated into the preservation of ciliary ultrastructure in vivo. Airway ciliary dysfunction and structural damage represent cardinal features of severe respiratory viral infections, including COVID-19 [51]. Therefore, by intratracheally delivering SARS-CoV-2 NSP13, a highly conserved helicase playing a key role in viral pathogenesis, we can effectively mimic key aspects of viral mucosal injury in vivo, thereby establishing a clinically relevant model of ciliary damage. Based on this model, we administered Kae (12.5, 25, or 50 mg/kg) via oral gavage and performed TEM of tracheal tissues to evaluate its therapeutic impact on airway ciliary ultrastructure. TEM analysis revealed pronounced structural abnormalities in NSP13-transfected mice, characterized by extensive ciliary loss, axonemal breakage, and irregular basal body positioning compared with the control group. Notably, Kae treatment substantially ameliorated these ultrastructural defects in a dose-dependent manner, with Kae-treated mice demonstrating increased apical ciliary density, properly aligned axonemal microtubules, and well-preserved basal regions with restored spatial relationships between basal bodies and axonemes (Figure 6B). These ultrastructural findings provide complementary evidence demonstrating that Kae effectively preserves and organizes ciliary architecture in vivo.

Together, these findings provide complementary cellular and ultrastructural evidence that Kae promotes microtubule recovery and preserves ciliary architecture, supporting the involvement of microtubule stabilization and basal body preservation in Kae-mediated ciliary elongation and repair in vivo.

## 4. Discussion

Ciliary dysfunction is increasingly recognized as a common pathological feature in a range of inherited ciliopathies and acquired diseases [2]. While previous studies have mainly focused on assembly and maintenance, our results identify Kae as a novel modulator that promotes ciliary elongation and restores ciliary length under impaired conditions, proposing axonemal microtubule stabilization as its underlying mechanism.

To rule out starvation-induced stress artifacts, we confirmed that Kae promotes ciliary elongation across normal physiological serum concentrations (10%, 5%, and 2% FBS) and diverse cell lines (RPE-1 and MRC-5). Furthermore, under 2% FBS conditions, evaluation at both early (3 h) and later (24 h) time points showed that Kae consistently restored ciliary length across three distinct ciliary injury models, demonstrating that its pro-elongation effect is evident as early as 3 h and persists at 24 h [47], indicating prompt modulatory effects on the axoneme (Figure S1D–I). Biologically, ciliogenesis involves early initiation (basal body docking and transition zone assembly) and subsequent elongation (IFT and microtubule assembly). In models with early initiation deficits (Ciliobrevin D inhibition or si*CEP290* knockdown), Kae fails to restore ciliation frequency, confirming it cannot bypass upstream blocks. Instead, by upregulating axonemal stabilizing factors (such as ACTUB) and membrane markers (such as ARL13B) [52,53,54], Kae selectively promotes the elongation of pre-existing or surviving axonemes.

It is quite intriguing that Kae maintains ciliary length-restoring activity across mechanically distinct injury models, including viral infection (NSP13) [55,56], motor blockade (Ciliobrevin D) [49], and genetic impairment (si*CEP290*) [57]. Although these models involve diverse upstream pathogenic insults, they converge on the disruption of axonemal integrity. Rather than correcting individual upstream defects, Kae reinforces conserved structural maintenance, as demonstrated by the restored localization of ARL13B, ACTUB, and IFT81. Notably, CEP290 depletion exhibited a particularly prominent ciliary elongation response. Because the C-terminal region of CEP290 directly interacts with microtubule structures, it is likely that the more robust microtubule network established by Kae facilitates the retention and anchoring of the residual CEP290 pool within the ciliary compartment [58,59].

Mechanistically, transcriptomic profiling revealed enrichment in microtubule dynamics and motor protein pathways, supported by the upregulation of DLGAP5 [17] and VASH2 [18], and downregulation of the ciliary suppressor KIF24 [19]. Nocodazole washout assays demonstrated that Kae pretreatment enhances cell-wide microtubule stability and reassembly dynamics. However, since whole-cell Western blotting measures total ACTUB without spatial isolation, and other tubulin PTMs like detyrosination associated with VASH2 were not systematically evaluated [60], whether acetylation is preferentially enriched in the axoneme requires further study. Crucially, because stable axonemal microtubules serve as the essential structural tracks for IFT, the analysis of IFT81 confirmed that Kae preserves IFT machinery functioning along the restored axoneme, linking microtubule stabilization to sustained ciliary elongation (Figure S3A,B) [61].

Regarding upstream regulation, accumulating evidence indicates that flavonoids modulate histone deacetylase (HDAC) activity [62,63]. Given that HDAC6 acts as a cytoplasmic deacetylase regulating α-tubulin stability [64,65], and that the Aurora A-HDAC6 pathway controls ciliary resorption [66], it is plausible that Kae counteracts HDAC6-dependent deacetylation to preserve acetylated axonemes. However, because HDAC6 activity and its requirement for Kae-mediated ciliary elongation were not directly examined in the present study, this possibility remains to be experimentally validated. Future studies using HDAC6 activity assays, genetic manipulation of HDAC6, or pharmacological approaches will be necessary to determine whether the HDAC6-α-tubulin acetylation axis contributes to the microtubule-stabilizing and ciliary effects of Kae. In addition, other potential mechanisms, including autophagy-mediated ciliogenesis, ciliary GPCR localization and membrane trafficking, and cilia-associated signaling networks, cannot be excluded and warrant further investigation to determine whether the structural rescue induced by Kae is accompanied by genuine functional recovery and to further clarify its relevance to cilia-associated disorders [67,68,69].

In summary, our study demonstrates that Kae specifically promotes elongation of endogenous cilia under physiological conditions and restores ciliary length across diverse models of ciliary dysfunction characterized by distinct stress mechanisms. Mechanistically, our results support axonemal microtubule stabilization as a common downstream mechanism for Kae-mediated restoration of ciliary length. Although the present study was limited to a qualitative assessment of the ultrastructural effects of Kae on ciliary microtubule architecture, future quantitative morphometric analyses may further characterize the structural changes associated with Kae-mediated ciliary restoration. By reinforcing ciliary architecture rather than inducing *de novo* ciliogenesis, Kae represents a previously unrecognized pharmacological approach for restoring dysfunctional cilia. Importantly, this mechanism-independent strategy may provide potential advantages over current approaches that primarily target individual genetic defects or disease-specific pathways, as stabilization of the conserved axonemal microtubule framework could enable broader applicability across genetically diverse forms of ciliary dysfunction. Hence, these findings expand the therapeutic landscape of cilia-directed interventions by identifying pharmacological restoration of ciliary length as a potential strategy for treating various inherited and acquired diseases associated with ciliary dysfunction.

## 5. Conclusions

In conclusion, Kae is identified as a novel pharmacological modulator of ciliary architecture that specifically promotes endogenous ciliary elongation in physiological context and restores defective cilia derived from diverse stress mechanisms. Kae improves microtubule-associated structural stability and reinforces axonemal integrity through a conserved mechanism underlying restoration of ciliary architecture in different models of ciliary dysfunction. Importantly, this mechanism-based strategy differs from existing approaches that largely target single genetic defects or disease-specific pathways, because it acts on a conserved structural framework shared among diverse ciliary disorders. Therefore, these findings position Kae as a promising microtubule-targeted pharmacological candidate for restoring ciliary structure and function.

## Data Availability

The original contributions presented in this study are included in the article/Supplementary Material. Further inquiries can be directed to the corresponding author(s).

## Supplementary Materials

The following supporting information can be downloaded at: https://www.mdpi.com/article/10.3390/cells15191806/s1, Figure S1: Kae promotes ciliary elongation under different FBS concentrations and attenuates ciliary defects across multiple models in ARPE-19 cells; Figure S2: Kae promotes ciliary elongation in MRC-5 and RPE-1 cells; Figure S3: Kae maintains IFT81 ciliary localization across multiple cilia-defect models.

## Abbreviations

The following abbreviations are used in this manuscript:

ACTUB	acetylated α-tubulin
ANKRD2	ankyrin repeat domain 2
ARL13B	ADP-ribosylation factor-like protein 13B
ARPE-19	adult retinal pigment epithelial cell line-19
ATP1A2	ATPase Na^+^/K^+^ transporting subunit alpha 2
BP	biological processes
BSA	bovine serum albumin
CC	cellular component
CENPE	centromere protein E
CEP290	collagen type IX alpha 2 chain
COL27A1	collagen type XXVII alpha 1 chain
CRYAB	crystallin alpha B
DAPI	4′,6-dia-midino-2-phenylindole
DEGs	differentially expressed genes
DLGAP5	disks large-associated protein 5
DYNLT4	dynein light chain tctex-type 4
EML6	EMAP like 6
GO	gene ontology
HRP	horseradish peroxidase
IFT	intraflagellar transport
ITGA2	integrin subunit alpha 2
Kae	Kaempferol
KEGG	kyoto encyclopedia of genes and genomes
KIF	kinesin family member
LBR	lamin B receptor
LMOD1	leiomodin 1
MRC-5	Medical research council cell strain 5
MF	molecular function
MYBPH	myosin binding protein H
MYL9	myosin light chain 9
MYO	myosin
MYOZ2	myozenin 2
NAV3	neuron navigator 3
NF-κB	nuclear factor kappa B
NRF2	nuclear factor erythroid 2-related factor 2
NSP13	non-structural protein 13
PCA	Principal component analysis
PFA	paraformaldehyde
REEP4	receptor accessory protein 4
RPE-1	hTERT-immortalized retinal pigment epithelial-1
SDC1	syndecan 1
STR	short tandem repeat
VASH2	vasohibin 2
