# Supplementary material for: Kaempferol Restores Cilia Length Through Axonemal Microtubule Stabilization
Source: Cells. 2026 Oct 3;15(19):1806. doi: 10.3390/cells15191806 (PMC13664028; doi:10.3390/cells15191806)
Supplement: Supplementary file 1 [file cells-15-01806-s001.zip › cells-4528183-supplementary.pdf]

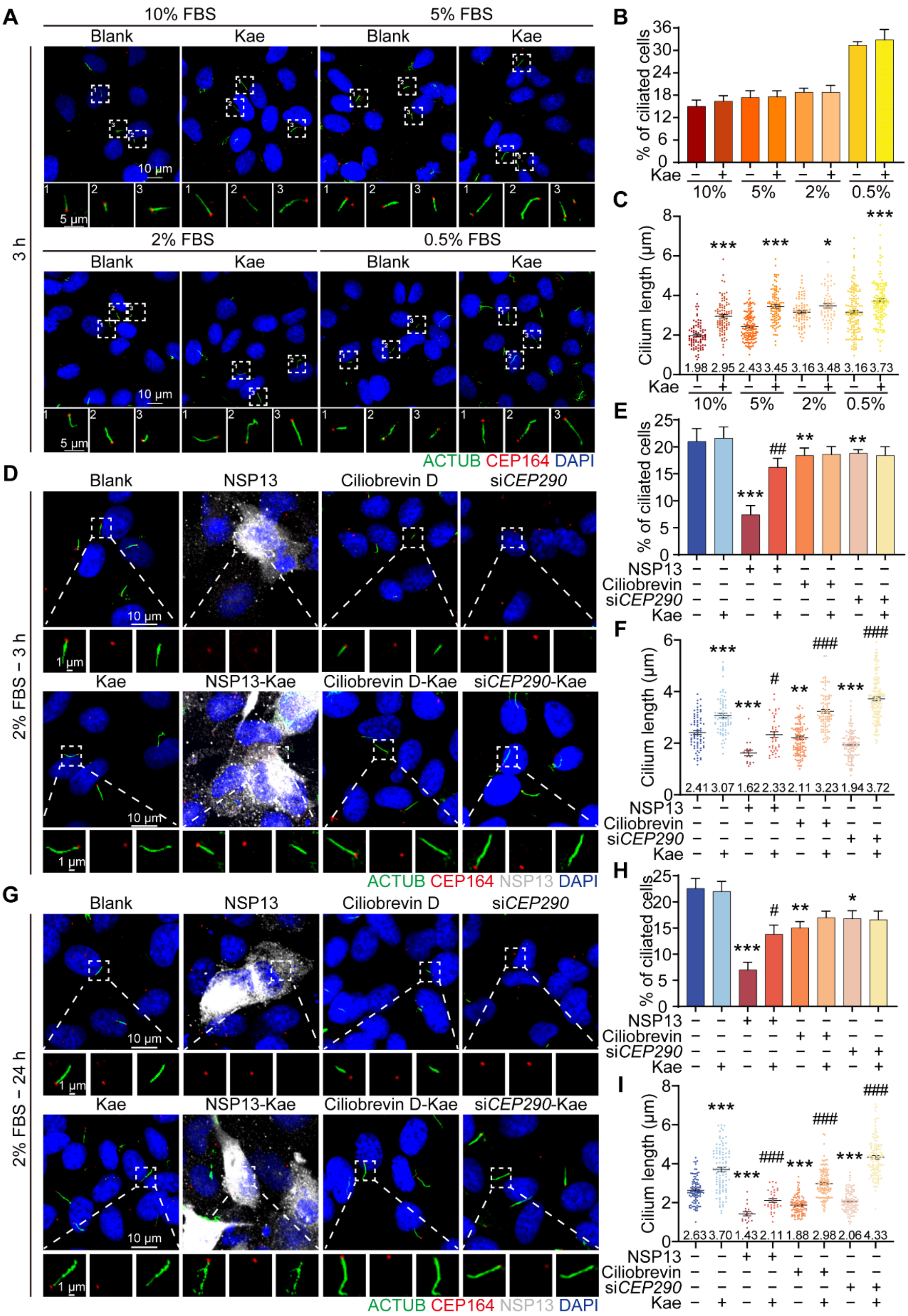

Figure S1. Kae promotes ciliary elongation under different FBS concentrations and attenuates ciliary defects across multiple models in ARPE-19 cells. (**A**–**C**) Representative IF images of cilia in ARPE-19 cells treated with Kae for 3 h under different serum concentrations (10%, 5%, 2%, and 0.5%). Cilia were stained with ACTUB (green) and CEP164 (red); nuclei were stained with DAPI (blue). Scale bars: 10 μm (upper panels) and 5 μm (lower panels). Quantitative analysis of cilia characteristics, including ciliation frequency (**B**) and ciliary length (**C**). Data are presented as mean ± SEM (Cilia length was measured across

10 fields of view, with approximately 20–30 cilia measured per field of view); **p* < 0.05, ****p* < 0.005 vs. Blank. (**D**–**F**) Representative IF images of ARPE-19 cells cultured under 2% FBS for 3 h and treated with Kae in the presence of NSP13-induced, ciliobrevin D-induced, or CEP290-deficiency cilia defect models, with blank control included. Cilia were stained with ACTUB (green), CEP164 (red), NSP13 (gray) and nuclei were stained with DAPI (blue). Scale bars: 10 μm (upper panels) and 1 μm (lower panels). Quantitative analysis of cilia characteristics, including ciliation frequency (**E**) and ciliary length (**F**). Data are presented as mean ± SEM (Cilia length was measured across 10 fields of view, with approximately 20–30 cilia measured per field of view); ***p* < 0.01, ****p* < 0.005 vs. Blank. #*p* < 0.05, ###*p* < 0.005 vs. Model. (**G**–**I**) Representative IF images of ARPE-19 cells cultured under 2% FBS for 24 h and treated with Kae in the presence of NSP13-induced, ciliobrevin D-induced, or CEP290-deficiency cilia defect models, with blank control included. Cilia were stained with ACTUB (green), CEP164 (red), NSP13 (gray) and nuclei were stained with DAPI (blue). Scale bars: 10 μm (upper panels) and 1 μm (lower panels). Quantitative analysis of cilia characteristics, including ciliation frequency (**H**) and ciliary length (**I**). Data are presented as mean ± SEM (Cilia length was measured across 10 fields of view, with approximately 20–30 cilia measured per field of view); ****p* < 0.005 vs. Blank. ###*p* < 0.005 vs. Model.

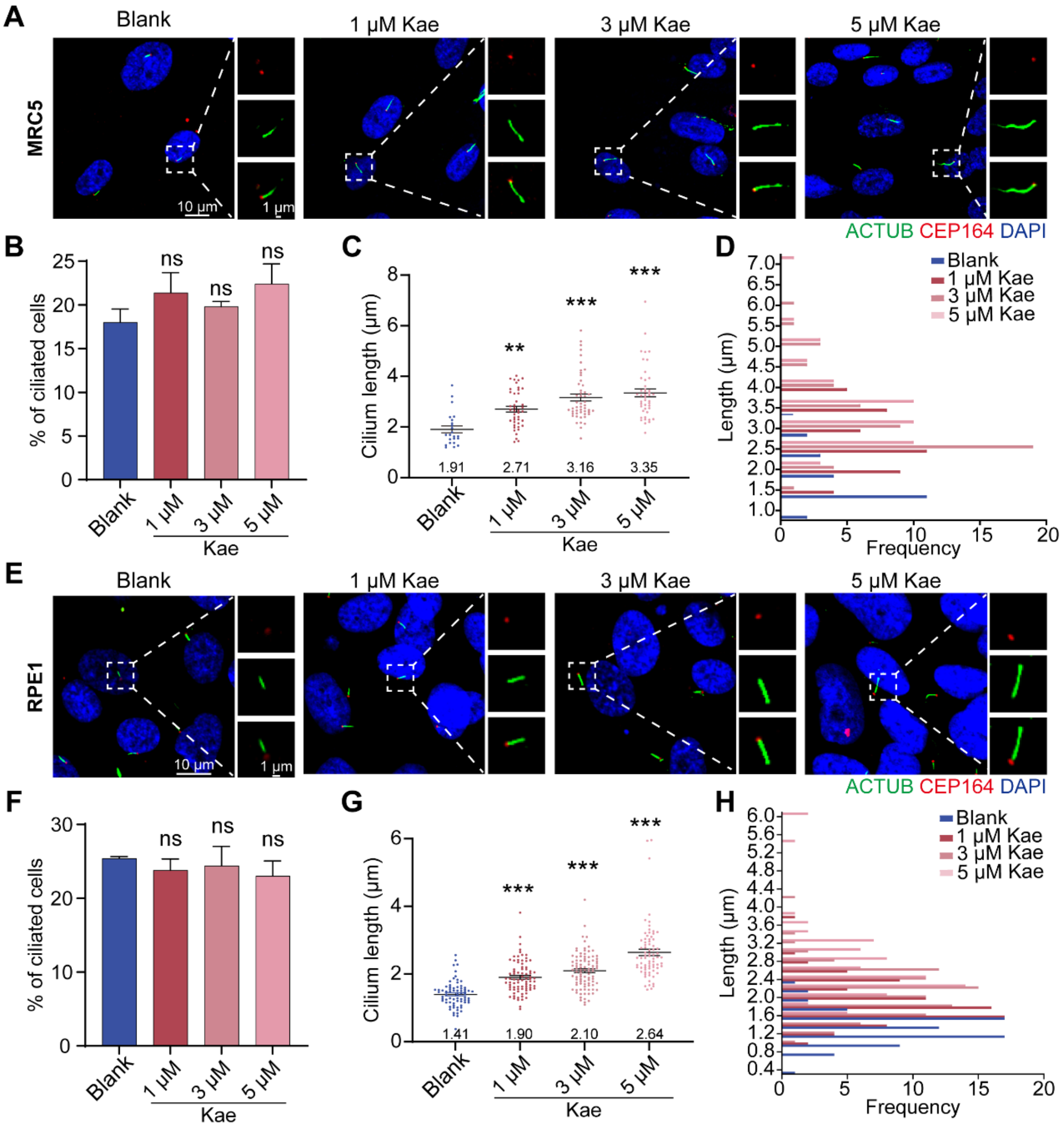

Figure S2. Kae promotes ciliary elongation in MRC-5 and RPE-1 cells. (**A**–**D**) MRC-5 cells were treated with DMSO or increasing concentrations of Kae (1, 3, and 5 μM) under serum-starved conditions to induce ciliogenesis. (**A**) Representative IF images of cilia. Cilia were stained with ACTUB (green) and CEP164 (red); nuclei were stained with DAPI (blue). Scale bars: 10 μm and 1 μm (magnified). Quantitative analysis of cilia characteristics, including ciliation frequency (**B**), ciliary length (**C**)and length frequency distribution (**D**). Data are presented as mean ± SEM (Cilia length was measured across 10 fields of view, with approximately 10–20 cilia measured per field of view); ns, not significant; $^{**}p <$ 0.01, $^{***}p < 0.005$ vs. Blank. (**E**–**H**) RPE-1 cells were treated with DMSO or increasing concentrations of Kae (1, 3, and 5 μM) under serum-starved conditions to induce ciliogenesis. (**E**) Representative IF images of cilia. Cilia were stained with ACTUB (green) and CEP164 (red); nuclei were stained with DAPI (blue). Scale bars: 10 μm and 1 μm (magnified). Quantitative analysis of cilia characteristics, including ciliation frequency (**F**), ciliary length (**G**)and length frequency distribution (**H**). Data are presented as mean ± SEM (Cilia length was measured across 10 fields of view, with approximately 10–20 cilia measured per field of view); ns, not significant; $^{***}p < 0.005$ vs. Blank.

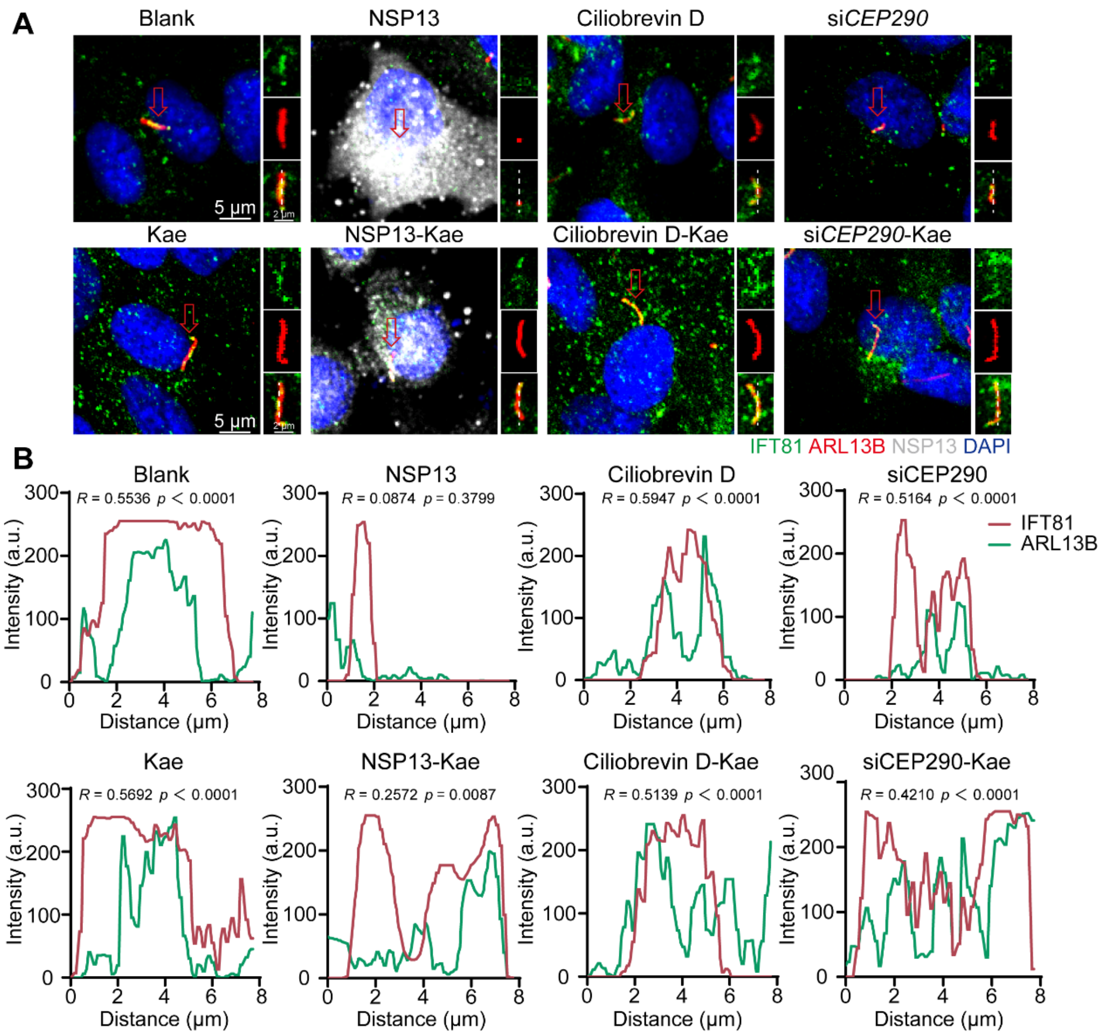

Figure S3. Kae maintains IFT81 ciliary localization across multiple cilia-defect models. (**A**) Representative IF images showing the localization of IFT81 (green), ARL13B (red), and NSP13 (gray) in cilia of ARPE-19 cells under NSP13-induced, ciliobrevin D-induced, or CEP290-deficiency conditions, with or without Kae treatment; nuclei were stained with DAPI (blue). Scale bars: 5 μm and 2 μm (magnified). (**B**) Line-scan analysis of IFT81 and ARL13B fluorescence intensity along the ciliary axis, showing their co-localization after Kae treatment.
